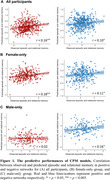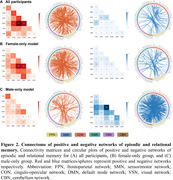# Connectome‐based predictions of cognition and sex differences in midlife individuals at risk for Alzheimer's Disease

**DOI:** 10.1002/alz70856_106501

**Published:** 2026-01-07

**Authors:** Qing Qi, Rory Boyle, Bolin Cao, Graciela Muniz‐Terrera, Ivan Koychev, Paresh Malhotra, John T O'Brien, Craig Ritchie, Brian Lawlor, Lorina Naci

**Affiliations:** ^1^ Global Brain Health Institute, Trinity College Dublin, Dublin, Ireland; ^2^ Trinity College Institute of Neuroscience, School of Psychology, Trinity College Dublin, Dublin, Ireland; ^3^ Penn Frontotemporal Degeneration Center, Department of Neurology, Perelman School of Medicine, University of Pennsylvania, Philadelphia, PA, USA; ^4^ Department of Neurology, University of Pennsylvania Perelman School of Medicine, Philadelphia, PA, USA; ^5^ Trinity College Dublin, Dublin, Ireland; ^6^ Department of Social Medicine, Ohio University, Athens, OH, USA; ^7^ Edinburgh Dementia Prevention, University of Edinburgh, Edinburgh, United Kingdom; ^8^ University of Oxford, Oxford, United Kingdom; ^9^ Imperial College London, Department of Brain Sciences, London, London, United Kingdom; ^10^ UK Dementia Research Institute, Care Research and Technology Centre, London, United Kingdom; ^11^ Department of Psychiatry, University of Cambridge School of Clinical Medicine, Cambridge, United Kingdom; ^12^ Scottish Brain Sciences, Edinburgh, Scotland, United Kingdom

## Abstract

**Background:**

Females generally show better memory performance, particularly in episodic memory, than males across the lifespan [1‐3]. However, two‐thirds of Alzheimer's disease (AD) cases occur in females, who experience more rapid cognitive decline and brain atrophy in the presence of AD‐related neuropathology [4]. The functional brain architecture underlying episodic and relational memory in middle‐aged individuals—and whether this differs by sex—remains poorly understood. This study aimed to identify the functional brain architecture associated with episodic and relational memory using a data‐driven approach, focusing on sex differences.

**Method:**

Resting‐state functional MRI data and neuropsychological assessments were obtained from 488 cognitively healthy individuals (316 F/172 M), aged 40‐59 years, from the PREVENT‐Dementia study. Connectome‐based predictive modeling (CPM) was used to identify functional brain networks related to episodic and relational memory across the entire cohort, within female‐only and male‐only subgroups. Model generalizability was evaluated using data from the Cambridge Center for Ageing and Neuroscience (Cam‐CAN) dataset.

**Result:**

CPM identified both positive and negative networks significantly associated with episodic and relational memory in the entire cohort (positive: r = 0.16, *p* < 0.001; negative: r = 0.10, *p* = 0.017; Figure 1A). These networks were particularly characterized by within‐network and between‐network connections involving default mode and cingulo‐opercular networks in the positive network (Figure 2A). Sex‐stratified analyses revealed distinct model performance: both positive and negative networks predicted episodic and relational memory in the female‐only group (positive: r = 0.18, *p*_‐corrected_ = 0.002; negative: *r* = 0.11, *p*_‐corrected_ = 0.048; Figure 1B), whereas only the negative network predicted episodic and relational memory in the male‐only group (positive: r = 0.01, *p*_‐corrected_ = 0.836; negative: r = 0.16, *p*_‐corrected_ = 0.048; Figure 1C) after Bonferroni correction. These results didn’t generalize to the external Cam‐CAN dataset.

**Conclusion:**

We identified brain networks underlying episodic and relational memory in middle‐aged individuals, revealing sex‐specific differences. These findings suggest potential sex‐specific mechanisms in memory‐related brain networks during midlife, which may contribute to differing trajectories of cognitive decline in aging and Alzheimer's disease. However, the lack of generalizability to an external dataset underscores the need for further validation in diverse populations.